# Loitering with intent—Catching the outlier vessels at sea

**DOI:** 10.1371/journal.pone.0200189

**Published:** 2018-07-12

**Authors:** Jessica H. Ford, David Peel, Britta Denise Hardesty, Uwe Rosebrock, Chris Wilcox

**Affiliations:** 1 CSIRO Data61, Castray Esplanade, Hobart, Tasmania, Australia; 2 CSIRO Oceans and Atmosphere, Castray Esplanade, Hobart, Tasmania, Australia; Aristotle University of Thessaloniki, GREECE

## Abstract

Illegal, Unreported and Unregulated (IUU) fishing activities pose one of the most significant threats to sustainable fisheries worldwide. Identifying illegal behaviour, specifically fishing and at-sea transhipment, continues to be a fundamental hurdle in combating IUU fishing. Here, we explore the use of spatial statistical methods to identify vessels behaving anomalously, in particular with regard to loitering, using the Indonesian Exclusive Economic Zone (EEZ) and surrounding waters as a case-study. Using Automatic Identification System (AIS) for vessel tracking, we applied Generalized Additive Models to capture both the temporal and spatial nature of loitering behaviour. We identified three statistically anomalous loitering behaviours (based on time, speed and distance) and applied the models to 2700 vessels in the region. We were able to rank vessels for individual and joint probability of atypical behaviour, providing a hierarchical list of vessels engaging in anomalous behaviour. While identification of irregular behaviour does not mean vessels are definitely engaging in illegal activities, this statistical modelling approach can be used to prioritise the allocation of enforcement resources and assist authorities under the United Nations Food and Agricultural Organization Port State Measures Agreement for management and enforcement of IUU fishing associated activities.

## Introduction

Illegal, Unreported and Unregulated (IUU) fishing activities pose a significant threat to sustainability in capture fisheries worldwide. IUU fishing by its nature is unregulated and unreported, without concern or incentive for sustainability; such activities can result in the depletion of fish stocks, loss of economic revenues, and reduction in local livelihoods.

IUU fishing activities can take many forms ranging from large foreign vessels stealing fish from the domestic waters of another country to small domestic operators failing to report catch rates accurately or those using gear for which they are not licensed. It also includes vessels that are engaged in activities that support IUU fishing behaviour, such as receiving and transferring catch, often called transhipment, and the refuelling of fishing vessels at sea. A recent United Nations report on crime in the fishing industry refer to all fishing activities (IUU fishing, at sea supply, refuelling and transhipments) as “fishing operations” [1]. These activities all play a role in enabling the crime, and in some cases support vessels may link networks of operators domestically and internationally.

In an effort to combat IUU fishing, the United Nations Food and Agricultural Organization (FAO), with member states, established the Agreement on Port State Measures which was adopted in 2009 [2], and went into force in 2016 [3]. The agreement aims to prevent, deter and eliminate IUU fishing through implementation of a suite of measures such as consistent transmission of location beacons to allow tracking of vessels, regular inspections in ports according to a common set of standards, and standardized electronic reporting of vessel, catch, monitoring, and inspection information among signatories [2].

While recent national and international efforts, such as the Port State Measure Agreement, give countries tools to address IUU fishing, identifying vessels among this large pool of actors remains a challenge. The FAO estimates that worldwide there are currently on the order of 690,000 fishing vessels greater than 12 meters in length, and 64,000 greater than 24 meters [4]. Understanding, and importantly, identifying anomalous behaviour, which could serve as an indicator of risk for illicit activity, to trigger inspections is a fundamental step to combating IUU fishing and other maritime issues. However, IUU fishing behaviours are variable and can be hard to detect. Developing a suite of tools that facilitate the identification of such behaviours will lead to increased opportunities and improved efficiency in the use of enforcement resources, both of which are fundamental components to preventing, deterring and eliminating IUU fishing.

Automatic Identification System (AIS) transmissions are currently the most broadly used and promising data for vessel tracking, as the system has global coverage and includes a large proportion of the ocean-going vessels [5]. Despite the availability of AIS data, however, there are several fundamental issues hampering their use in addressing IUU fishing. First, handling these data requires a specialized platform that can collect the data, manage the data, provide tools for analysis, and facilitate the visualization of the outputs. This is generally carried out using a mix of proprietary software and geospatial databases. However, neither geospatial databases nor available proprietary software is particularly useful for implementing analytical tools, beyond basic geospatial manipulation. Second, much of the focus in the surveillance community is on real-time or near real-time use of the data, as opposed to either retrospective or predictive analysis. Thus far, to detect anomalous behaviour researchers have applied clustering algorithms to summary statistics of vessel tracks, such as: turning rate, number of turns and stops [6], low-likelihood trajectories [7]; and spatial clustering to identify stationary areas of maritime vessel traffic [8].

An alternative approach to the analysis of AIS data is to use spatial models of vessel movement to summarize normal or typical behaviour, and then analyse individual vessel’s movements to look for anomalies that depart from this typical behaviour. This approach has the advantage of incorporating location, a key element correlated with the behaviour of vessels. Such models can be used to identify specific vessels behaving anomalously, or to identify ‘hotspots’ where such behaviour occurs (for example, at EEZ boundaries or unpatrolled areas). They can also be used predictively, to identify vessels classified as behaving anomalously in real time as the AIS data comes in.

In this paper we first describe the model, and then demonstrate its application in an area near the joint border of Indonesia, Papua New Guinea and Australia, an area linked with a high intensity of both legal and, at times, illegal fishing activity. Finally, we demonstrate the use of the method to produce a list of vessels with probabilistic ranking of anomalous behaviour to which further investigation can be applied.

## Methods

### Study site

We utilize Automatic Identification System (AIS) data in the area where the EEZ boundaries of Indonesia, Papua New Guinea (PNG) and Australia meet in the Arafura Sea. The AIS is an anti-collision system implemented internationally for vessels over 300 gross tons. Since its implementation in 2002, AIS use has expanded to include smaller vessels and previously exempted vessel types, such as fishing vessels.

There are a wide range of at-sea activities occurring in the study area. Several major shipping lanes pass through the region. There are a number of major ports for each of the littoral countries. Illegal fishing activity has also been a major feature in the region, with reports of significant activity historically [9]. We used data from January 2015 through to May 2016 for this case study region, with 2.6 million unique AIS records across all 2700 vessels–regardless of vessel type (i.e. tanker, bulk carrier, fishing, cargo, military, pilot). Data were obtained from the Australian Maritime Safety Authority.

To avoid the inclusion of spurious tracks in our model, we removed any track segments with calculated speed more than 60 knots. Rules for data cleaning associated with speed cut offs and track segment lengths can be adjusted in the model, depending on the application and context.

### Indicators

We calculated three response variables (herein referred to as *indicators*) from the AIS data for our analysis: time spent in a location, distance travelled and average vessel speed in a location. These indicators were used as they provide measureable, quantifiable movement summaries of AIS data for an individual vessel. Other metrics which go beyond loitering behaviour and relate more to general abnormal behaviour (e.g. typical heading, boat class, size, nations registered) could also be considered. Inclusion of additional covariates such as ship type is possible, however such data derived from AIS messages are often unreliable and incomplete and so have not been included in this analysis.

Given a data set of AIS locations for a specific vessel, we assume track segment S_*i*_ is the *ith* track segment joining the points between time *i* and *i+1*, that is between the recorded locations *X*_*i*_ and *Y*_*i*_ and *X*_*i+1*_ and *Y*_*i+1*_, where *X* is longitude, and *Y* is latitude. Each track segment *S*_*i*_, is divided in to *n*_*i*_ smaller intervals of given interpolation distance *d* (or time *t*) based on overall distance travelled (or time in seconds). That is, if given track segment *S*_*i*_ is 1500 meters, and interpolation distance *d* is 10 meters, *n*_*i*_ for track segment *S*_*i*_ is 150.

We grid the study area into cells of specified size (for example 0.25 degree by 0.25 degree) and then for each cell, *C*_*i*_, we find the number of smaller segments of each track segment *S*_*i*_ which lie continuously within the boundaries of cell *C*_*i*_. If a vessel leaves the cell and re-enters this is recorded as two separate summary statistics. Given the calculation within a cell, if the behaviour of an indicator occurs along the boundary of two cells, this behaviour may be missed depending on how the track is split.

For calculation of distance travelled in each cell, we find the sum of all smaller intervals *n*_*i*_ for each track segment *S*_*i*_ in the cell *C*_*i*_. The sum of the smaller intervals is multiplied by the specified interpolation distance, which determines *n*_*i*_, for example 10 meters.

The same method was applied for time spent in cell, where time for track segments (that is time between *t*_*i*_ and *t*_*i+1*_) were divided in to smaller intervals based on specified interpolation value, here we used 30 second track segments.

Using this interpolation method, we calculated three main track summaries of anomalous loitering behaviour: time spent in a cell (seconds); distance travelled in a cell (meters); and average speed in cell (knots). The location of each cell was recorded as the latitude and longitude at the centre of the cell.

### Model

We used Generalized Additive Models (GAM) which allow us to capture both spatial and temporal patterns of vessel behaviour in the study region [10]. The GAM employed allowed us to identify normal behaviour in vessel movement using three different indicators: time spent in an area, average speed in an area, and distance travelled in an area. Given the ‘normal’ behaviour at a given spatial location and time, we can then predict the likelihood of a vessel’s observed movements. Loitering does not imply illegal activity, but non-normal behaviour can be used as a risk factor to flag for vessels potentially engaging in illegal activity. For a hypothetical example, say there is a cell where the majority of vessels travel slowly due to navigational issues but are just traversing the cell, and then consider a vessel that also travels slowly but does not simply traverse the cell but stays in the cell. In this case the vessel would be flagged as having abnormal time in cell but not abnormal speed. This does not imply that the vessel is illegal, but flags it for unusual behaviour–why was it there and what was it doing? For example, was the longer time due to mechanical problems or was the vessel in an area known for IUU fishing and thus potentially involved in IUU fishing activity, either fishing, transhipment or refuelling. Regardless, the flag does not indicate illicit activity, but instead highlights the need for further investigation. These statistical models are able to represent relationships between an indicator, e.g. vessel speed, and a predictor variable, e.g. location, using a mix of parametric terms as in traditional regression and nonparametric terms made of a combination of smooth functions [10]. These models are particularly useful for representing spatial patterns, as they allow fitting of a smoothly varying spatial component–in essence, the statistical version of a map–in a context where other parametric and non-parametric terms can also be included. We used the *gam* function in the *mgcv* package [11] in the R statistical language [12].

We used a Tweedie distribution to model each of the three indicators of loitering behaviour: time spent in area, distance travelled in area, and average speed in area, due to the long tails in the distributions of the derived variables. We fit three individual models for each of the indicators of loitering behaviour.

The final models used were: loitering indicator ~ s(lat,lon), where s(.) represents a smoothed interaction term for Latitude and Longitude, using a soap film smooth to account for coastal boundaries in the region [13]. Based on these models, we then predicted the probability, given the spatial surface, of each loitering indicator (speed, distance, and time) for all data points.

### Anomalies and ranks

Predictions were found for all observations in the data, and loitering indicators with probabilities below 1e-6 were used to identify high risk, irregular behaviour. To rank vessels, the first percentile (*p*_*1*_) of the distribution for each vessels’ loitering anomalies was compared to the location of all other vessels *p*_*1*_ on the combined distribution. The combined product of these three values was used to create an overall rank for the three loitering anomalies. Using this approach, individual or any joint combination of anomalies can be determined. This results in list of vessels, hierarchically ranked for high risk irregular behaviour.

## Results

### Evaluating the spatial distribution of speed, time and distance metrics

**Fig 1:**
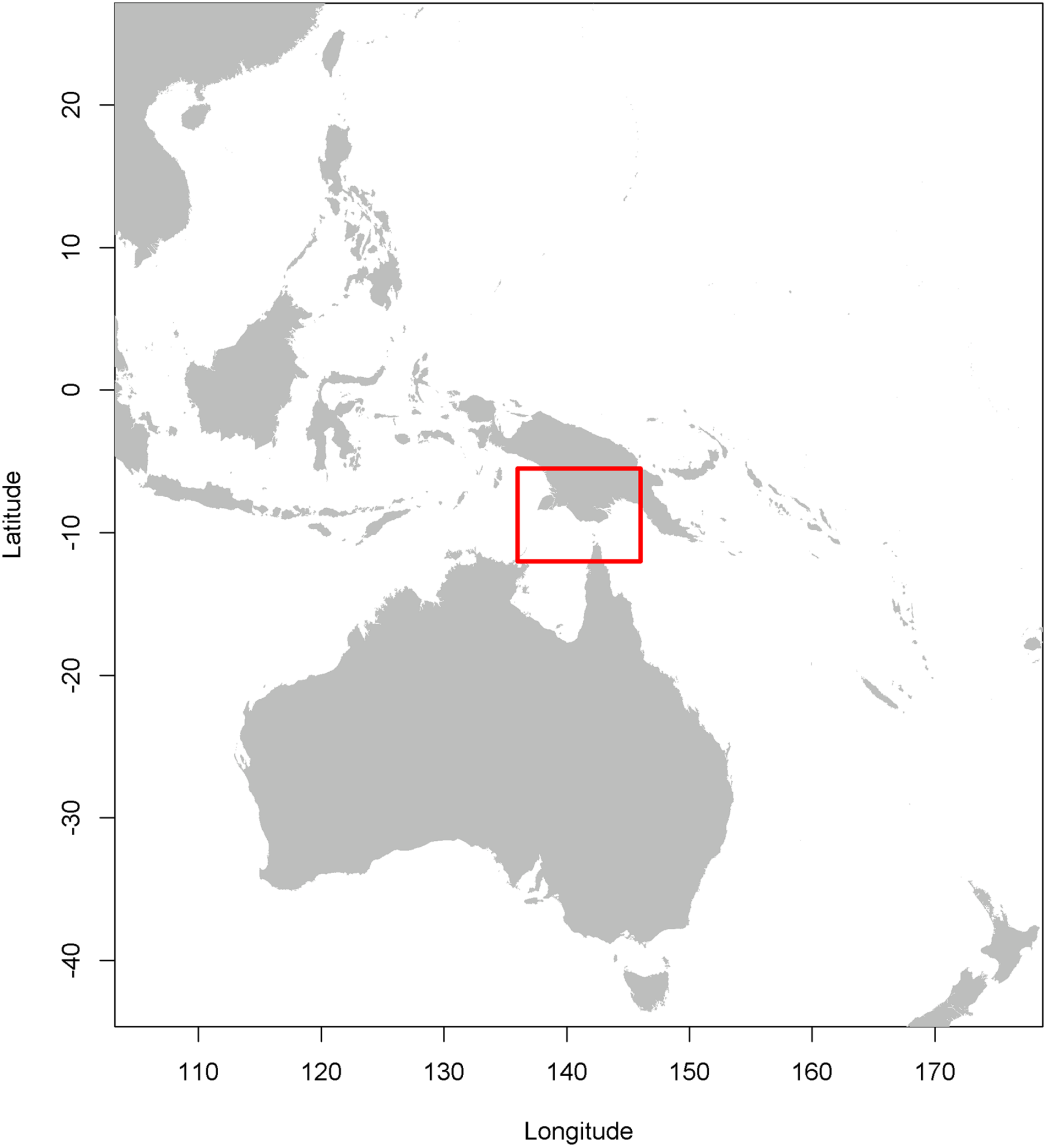
Geographic study area. The red box indicates the case study region.

**Fig 2:**
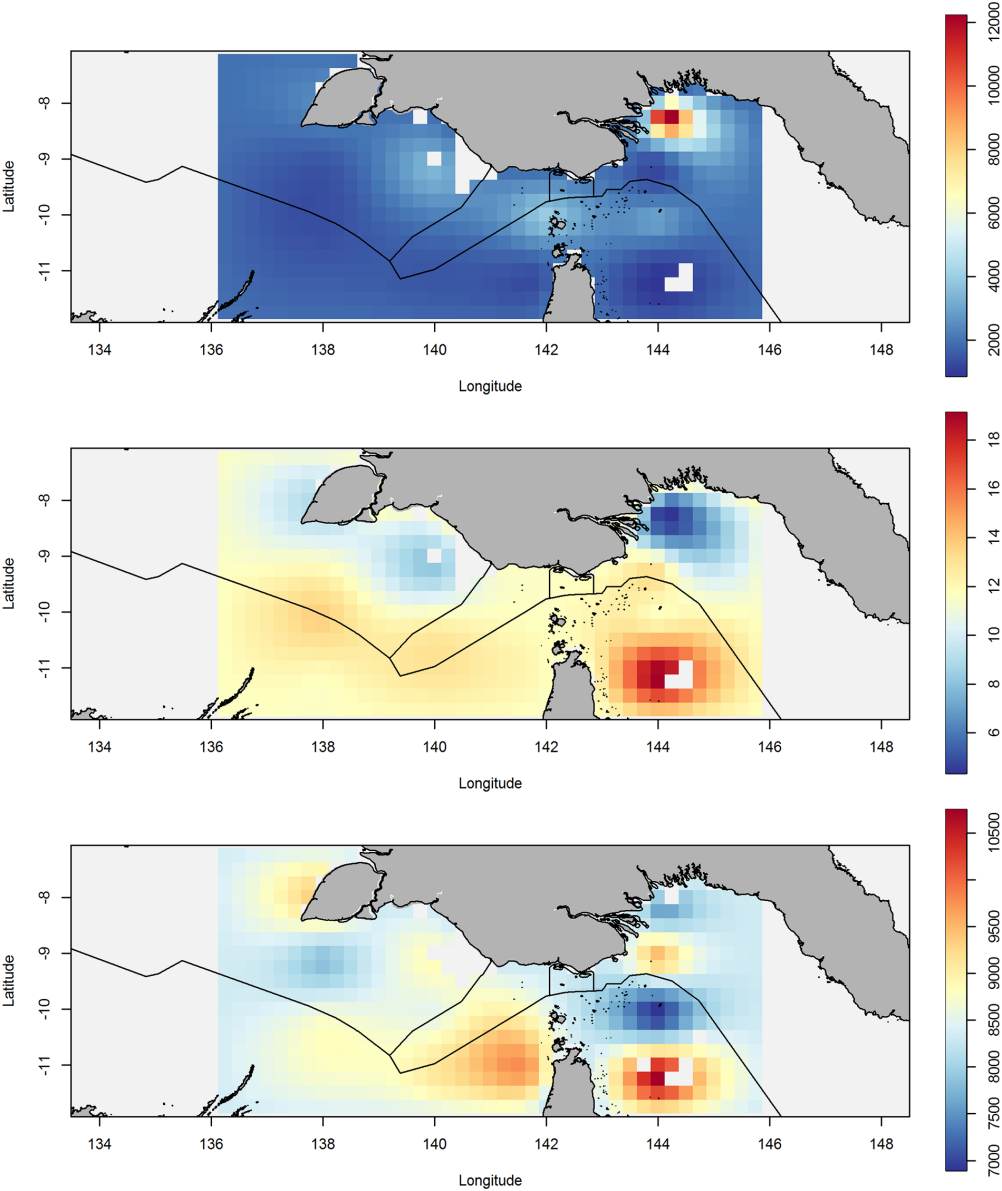
**2a**) Prediction surface for time loitering indicator. Scale shows predictions in seconds for time spent in cells. **2b**) Prediction surface for speed loitering indicator. Scale shows predictions in knots for average speed in cells. **2c**) Prediction surface for distance loitering indicator. Scale shows predictions in meters for distance travelled in cells.

We fitted the GAM to data in the Arafura Sea boundary region between Indonesia, PNG, and Australia (Fig 1). Spatial variation across the region is evident for each of the three loitering indicators (Fig 2a–2c). For the time indicator, cells around land, such as major ports, have longer time spent in cell compared to those further from shore (Fig 2a). The standard errors for the models indicated a good fit, and as expected, regions of low data density displayed higher uncertainty compared to regions with higher densities.

### Detecting anomalies in the metrics and building an integrated score

**Fig 3:**
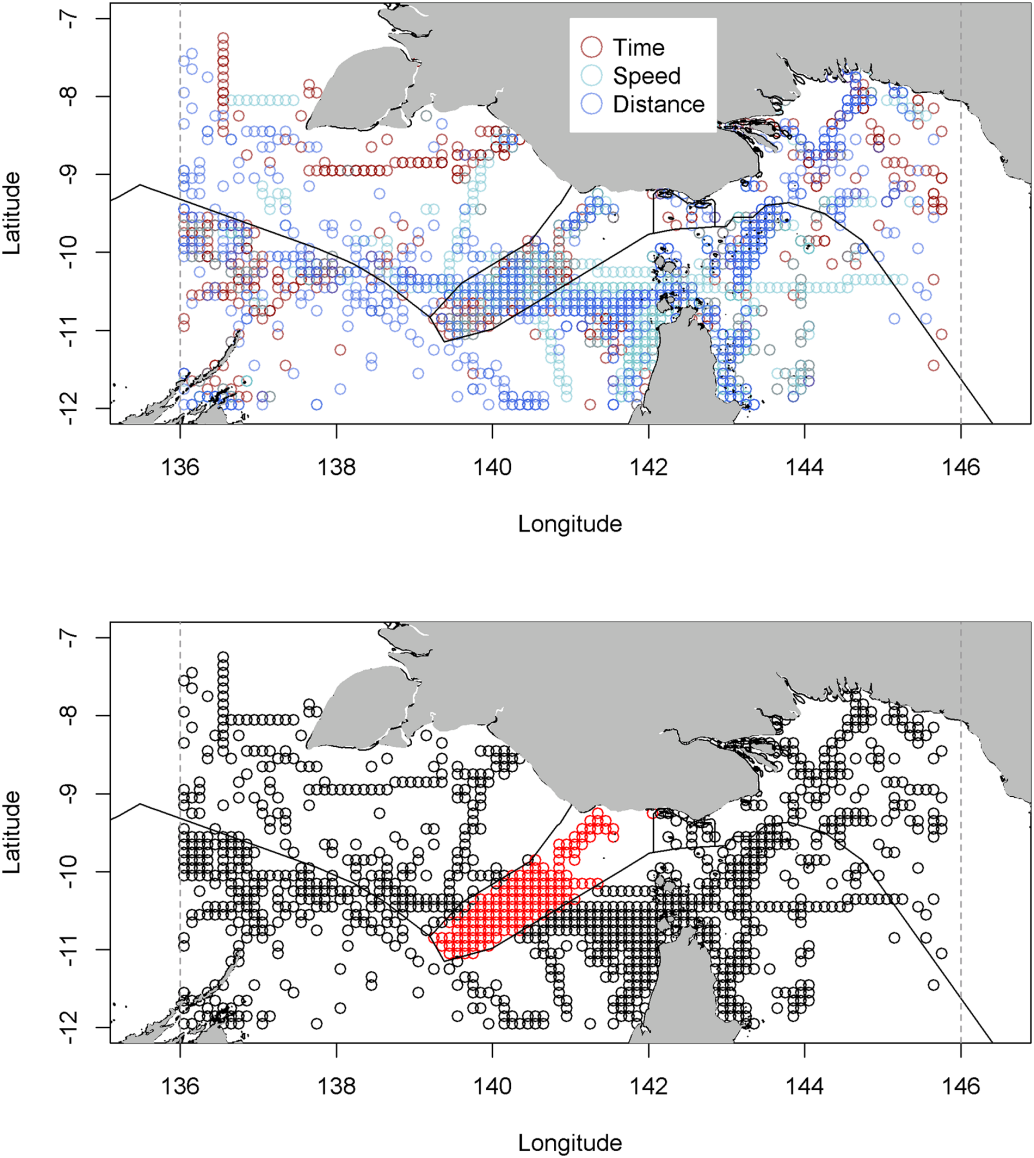
**3a**) High risk anomalies for time, speed and distance. **3b**) All high risk anomalies. Anomalies in the trination region bounded by Australian, Indonesia and Papua New Guinean are highlighted red.

We used the fitted Tweedie distributions from the GAMs to calculate a probability of an observed value for the three loitering indicators (time, speed, distance) for each observation in our dataset. High risk indicators of irregular behaviour were identified as those with probabilities below 1e-6. These high risk anomalies correspond to the top ½ to 1 percent of all anomalies (see Fig 3a).

**Fig 4:**
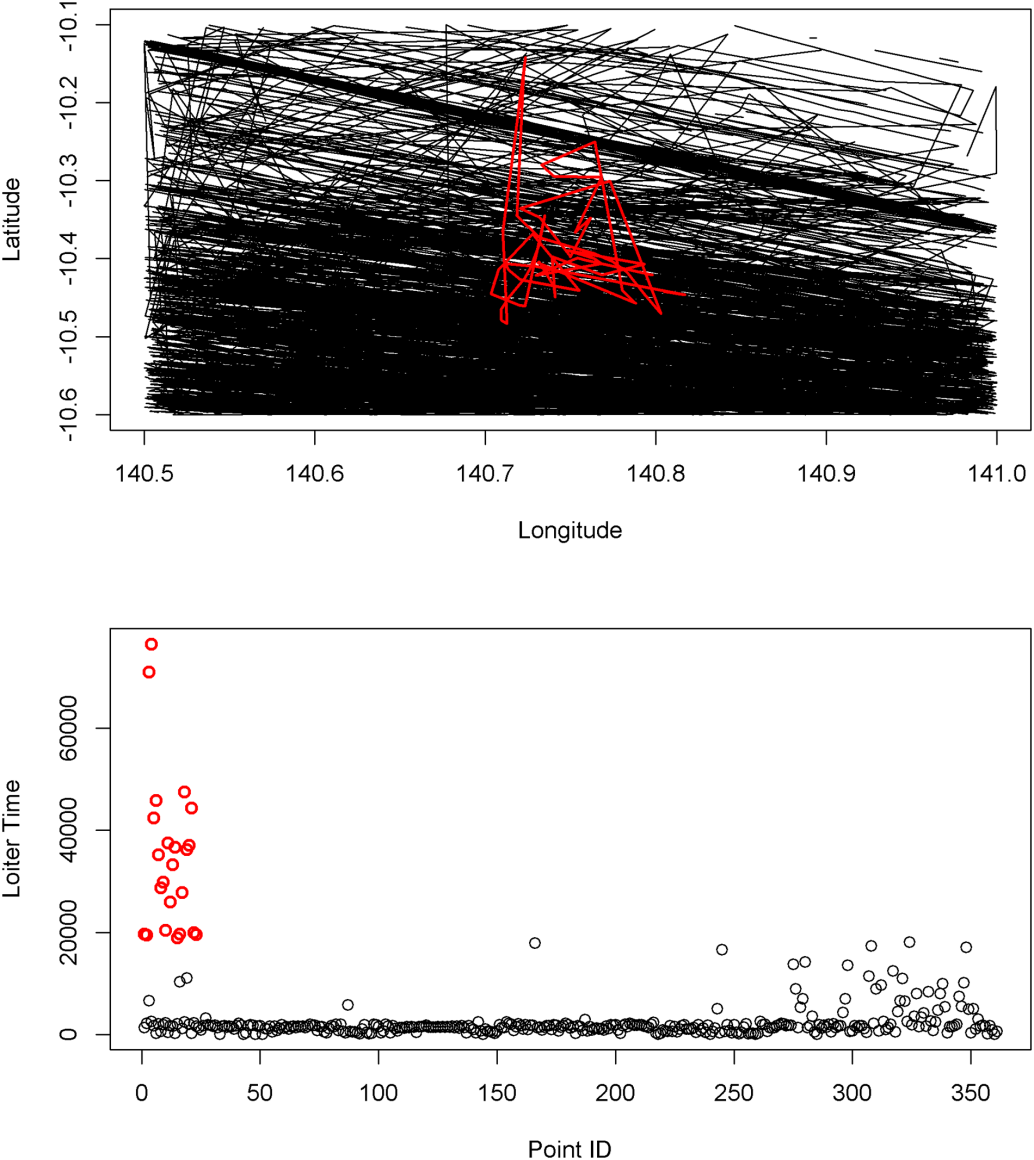
**4a**) An example of an anomalous high risk track (in red) identified using the distance indicator, and all other tracks in the 0.5 degree cell (black). Note the vessel track in red moves across latitude rather than longitude, a discernible difference from other vessels transiting in the region. **4b**) Depicts the time spent in the same 0.5 degree cell, for each track segment shown in 4a, with the anomalous vessel (red) and all other vessels (black) highlighted.

Tracks identified by our statistical algorithm clearly stand out when plotted against other vessel tracks in the region (Fig 4). For instance, comparing distances travelled, the majority of normal tracks in a 0.5 degree cell in our study region might make a linear transit across the cell, while an anomalous vessel moves much further latitudinally within the cell (Fig 4a). Similarly, comparing time spent in the same cell, anomalous vessels might spend a much longer time in the cell (>5.5 hours), while other vessels make relatively short transits in and out of the cell (Fig 4b).

### Identifying hotspots and vessel characteristics for anomalous vessels

We ranked vessel anomalies using calculations that took into account both individual and joint metrics. Within the top 100 vessels, using a joint metric (the product of all individual lower *p*_*1v*_), almost half the vessels were in the class types tug, pilot, military, or fishing vessel. Notably, within and around Australian waters, these occur in the Northern offshore Islands (north of Cape York) and around the tri-nation EEZ boundary known as the ‘dogleg’. The independent scores show some clear spatial patterns—with anomalies in ports and along EEZ boundaries (Fig 3a). Across the region, 77 vessels registered with one or more high risk event in each of the loitering indicators.

The dogleg is an area known for activities associated with IUU fishing [14]. Taking a subset of all anomalies within the dogleg (see Fig 3b)—54 vessels were identified with at least one low probability anomaly in this boundary. These vessels included 31 cargo, tug and various supply vessels, two military vessels, 14 vessels possibly engaged in supporting IUU fishing and 7 foreign fishing vessels (flag nations other than Australia, Indonesia and PNG).

The frequency of loitering behaviour can be used as another measure of irregular activity. In this subset of 54 vessels, the fishing vessels had higher frequency of anomalous events compared to the cargo and tug vessels. The time metric was the most sensitive to potential IUU fishing behaviour: of the 20 vessels in the dogleg area that were flagged with high-risk time anomalies, eight were foreign fishing vessels, and seven appeared to be potential reefer and refuelling vessels.

The majority of the high-ranked vessels across the region were comprised of a small subset of vessel types, including law enforcement and patrol vessels, tug and pilot vessels and fishing and fishing support vessels (almost half of the top 100 vessels were in one of these three classes). These different types of vessels were detected as having anomalous behaviour in particular locations, with tug and pilot vessels near ports, but not along EEZ boundaries. Fishing vessels were much more likely to be identified as anomalous near EEZ boundaries.

## Discussion

Given the large number of vessels at sea and limited resources available to manage high seas fishing, prioritizing vessels on which to focus for the most efficient use of resources is a key step in the challenge of reducing IUU fishing activities. However, identifying illegal activities on the water is difficult as the actions are hard to observe. Using risk-based approach, such as presented here, will be critical to improving enforcement opportunities as they can provide indications of potential illicit behaviour. We have presented one approach for identifying anomalous movement behaviour. A comprehensive risk framework, which shifts the focus from general anomalous maritime activity towards IUU fishing associated activity, can be achieved by combining the method presented here, with additional IUU fishing associated risk-based models, for example gaps in AIS activity and proximity to fishing locations.

Using spatial statistical models, we identified anomalies in behaviour using three different indicators of loitering behaviour: time spent in an area, average speed in an area, and distance travelled within an area. Using fitted models, we successfully calculated the expected probability of all three loitering indicators for each of 2700 vessels in the Arafura Sea region and identified a subset of 54 vessels that warranted further attention.

Each of the individual attributes that identify a vessel as anomalous are important; they are informative in their own right and as an ensemble. For instance, a vessel may exhibit anomalous behaviour with slow speed, but may not necessarily for distance. If a vessel enters an area, and loiters such that it remains somewhat stationary, then the vessel will be identified based on speed in the area if the remaining vessels are transiting through. However, a vessel may also enter a cell, follow the shipping route, but take longer and travel slower–thus it may be flagged for time spent, and low speed, but not distance. In fact, this exact behaviour has been noted by fisheries managers in the Pacific, where cargo vessels travelling along established shipping routes make short stops to receive fish illegally.

One potential issue with other approaches to model anomalous behaviour is that previous models often treat each spatial cell as independent, and do not take in to account neighbouring cells [15]. This is likely unrealistic, as vessel behaviours in adjoining locations are likely similar due to their proximity alone and could lead to spurious estimates in cells with few observations, or the need to exclude those cells altogether. One notable advantage of the spatial modelling approach we present here is that we do not treat neighbouring cells as independent, since by their nature GAMs allow spatial dependency. Hence, if there is little or no data at a certain location, strength is gained by incorporating information from nearby locations. Our approach also differs from some other anomaly detection algorithms that work with whole vessel tracks (e.g. [7]). As we focus on the spatial pattern in a location across vessels, instead of the typical vessel pattern across space, our model can readily incorporate additional variables in the temporal or spatial domain, along with characteristics of individual vessels.

The model we used is based on an explicit probability distribution. Since analyses are based on an established probability distribution, the values can be compared or combined in a transparent manner following the rules of probability and can carry through any uncertainties around their rankings. The outcome of a hierarchical list is an overall rank of anomalous loitering behaviour for which further investigation is possible, with the added opportunity to target limited resources for best management or enforcement outcomes. Furthermore, such a list can be compared to other types of data to identify other behaviours which might indicate illicit activity, such as proximity to other vessels.

The model we applied is computationally efficient and can be easily extended to include other summary variables (such as turning, length, beam etc.). The model does not require a known training set, hence it can be applied readily to identify geographic regions of interest or hotspots where unusual behaviour occurs, such as at EEZ boundaries, or unpatrolled areas. Furthermore, the model can be used retrospectively to identify vessels with historical patterns that stand out, in real time to identify vessels with irregular behaviour that might warrant interdiction, or prospectively to predict the future location or identity of vessels engaged in suspicious behaviour.

Based on the consensus of a workshop of maritime surveillance practitioners, our anomaly detection algorithms provide an important tool to assist port authorities under the FAO Port State Measures Agreement to identify factors associated with direct IUU fishing activities, as well as transhipment, high-seas refuelling, or other anomalous behaviours. While we applied the models developed to IUU fishing associated activities, the method is broadly applicable to other maritime and border security issues such as trafficking in persons, smuggling of migrants and trafficking of illicit drugs. Monitoring anomalous behaviour by their vessels is also likely to be a key interest for flag states, as pressure is increasing for them to fullfill their “due diligence” responsibilities under the Law of the Sea [16] to ensure their vessels are not involved in IUU fishing or other illegal activities.
